# Delivery of size-controlled long-circulating polymersomes in solid tumours, visualized by quantum dots and optical imaging *in vivo*

**DOI:** 10.1080/13102818.2014.984894

**Published:** 2014-11-26

**Authors:** Rumiana Bakalova, Desislava Lazarova, Biliana Nikolova, Severina Atanasova, Genoveva Zlateva, Zhivko Zhelev, Ichio Aoki

**Affiliations:** ^a^ Diagnostic Imaging Program, Molecular Imaging Center, National Institute of Radiological Sciences, Anagawa, Chiba, Japan; ^b^ Department of Physics, Biophysics and Roentgenology, Faculty of Medicine, Sofia University “St Kliment Ohridski”, Sofia, Bulgaria; ^c^ Department of Electroinduced and Adhesive Properties, Institute of Biophysics and Biomedical Engineering, Bulgarian Academy of Sciences, Sofia, Bulgaria; ^d^ Department of Medicinal Chemistry and Biochemistry, Faculty of Medicine, Trakia University, Stara Zagora, Bulgaria

**Keywords:** drug-delivery systems, nanoparticles, polymersomes, quantum dot, fluorescent imaging, cancer

## Abstract

The present study was designed to investigate whether poly-ion complex hollow vesicles (polymersomes), based on chemically modified chitosan, are appropriate for passive tumour targeting in the context of their application as drug carriers. The experiments were performed on colon cancer-grafted mice. The mice were subjected to anaesthesia and injected intravenously with water-soluble nanoparticles: (1) QD^705^-labelled polymersomes (average size ∼120 nm; size distribution ∼10%) or (2) native QD^705^. The optical imaging was carried out on Maestro EX 2.10 In Vivo Imaging System (excitation filter 435–480 nm; emission filter 700 nm, longpass). In the case of QD^705^, the fluorescence appeared in the tumour area within 1 min after injection and disappeared completely within 60 min. A strong fluorescent signal was detected in the liver on the 30th minute. The visualization of tumour using QD^705^ was based only on angiogenesis. In the case of QD^705^-labelled polymersomes, the fluorescence appeared in the tumour area immediately after injection with excellent visualization of blood vessels in the whole body. A strong fluorescent signal was detected in the tumour area within 16 hours. This indicated that QD^705^-labelled polymersomes were delivered predominantly into the tumour due to their long circulation in the bloodstream and enhanced permeability and retention effect. A very weak fluorescent signal was found in the liver area. The data suggest that size-controlled long-circulating polymersomes are very promising carriers for drug delivery in solid tumours, including delivery of small nanoparticles and contrast substances.

## Introduction

The advent of nanoparticle-based drug delivery systems (nano-DDS) opens new pathways to understanding physiology and pathophysiology at the nanometre scale. Target-specific nano-DDS can be used to deliver higher local concentrations of drugs to the region-of-interest (ROI) and magnify therapeutic effects. In the last years, the main efforts are directed to the development of nano-DDS for target-specific image-guided drug delivery in cancer, using different imaging techniques (magnetic resonance imaging, positron-emission tomography, optical imaging, etc.).[1–3] Selective disposition of nanocarriers into the target tissue is an essential issue in drug delivery. In the last several years, the critical size of nanocarriers (150 nm), discriminating the permeability into normal and tumour tissues, was determined by the use of size-tunable, polyion complex hollow vesicles (polymersomes) as a ruler.[1,4,5] Polymersomes are capable of encapsulating hydrophobic and hydrophilic drugs and they can be surface functionalized for target-selective drug delivery and delivery of imaging probes. Their polymeric membrane potentially offers a protective barrier to proteins, peptides, DNA and RNA fragments against deleterious factors that may be present in the biological environment.[1]

To evaluate the impact of polymersomes (made by different polymer matrices) as drug carriers, it is necessary to investigate their pharmacodynamics *in vivo* and especially the possibility to deliver them into the target tissue. One of the most preferred targets is cancer due to the efforts to develop highly specific therapeutic strategies with minimal side-effects. The polymersomes are usually labelled by different contrast agents and their pharmacodynamics was verified *in vivo* by optical imaging, magnetic resonance imaging, positron-emission tomography and multimodal imaging.[6–8]

Semiconductor quantum dots (QDs) are one of the most appropriate fluorescent markers for deep-tissue optical imaging of pharmacodynamics of polymersomes in living organisms and their ability to penetrate into the tumours.[9,10] In the last 10 years, QDs have been recognized as a new type of optical imaging contrast agents because of their unique spectral properties and many advantages over traditional organic fluorophores.[11,12] The following characteristics distinguish QDs from the commonly used fluorophores: high quantum yield (more 50% versus 15%–50% for standard organic dyes); high molar extinction coefficients (in the order of 0.5–5 × 10^6^ M^−1^ cm^−1^, which is ∼10–100 times larger than those of traditional organic dyes – 5—10 × 10^4^ M^−1^ cm^−1^); broad absorption spectra with narrow, symmetric photoluminescence spectra (full-width at half-maximum ∼25–40 nm) spanning the ultraviolet to near-infrared(NIR); low life-time-limited emission rates for single QDs (∼5–10 lower than those of single organic dyes), because of their longer excited state life-times (20–50 ns); large effective Stokes shifts; high resistance to photobleaching (several thousand times more stable than organic dyes); and exceptional resistance to photo- and chemical degradations, which makes them appropriate for tracking studies over a long period of time.[12] All these characteristics make QDs brighter fluorescent probes (10–20 times brighter than conventional organic dyes) under photon-limited *in vivo* and *in situ* conditions, where light intensities are severely attenuated by scattering and absorption.

These unique optical properties of QDs can be used to optimize the signal-to-background ratio, to improve the sensitivity of fluorescence detection and to increase the quality of fluorescent deep-tissue imaging *in vivo*.[13,14] Moreover, the size-tunable fluorescence emission and the broad excitation spectra of QDs make it possible to use them in multiplexed fluorescent analyses.[12–17] Single QDs can be observed and tracked for up to a few hours with optical (fluorescent) imaging systems, fluorescence confocal microscopy, total internal reflection microscopy, or basic wide-field epifluorescence microscopy and single-molecule microscopy. QDs are also excellent probes for two/multi-photon confocal microscopy because they are characterized by a large absorption cross-section.[12–17]

The present study was designed to investigate the passive delivery of size-controlled long-circulating polymersomes in solid tumours on experimental animals, visualized by quantum dots and optical imaging *in vivo*.

## Materials and methods

### Chemicals

**Figure 1.**
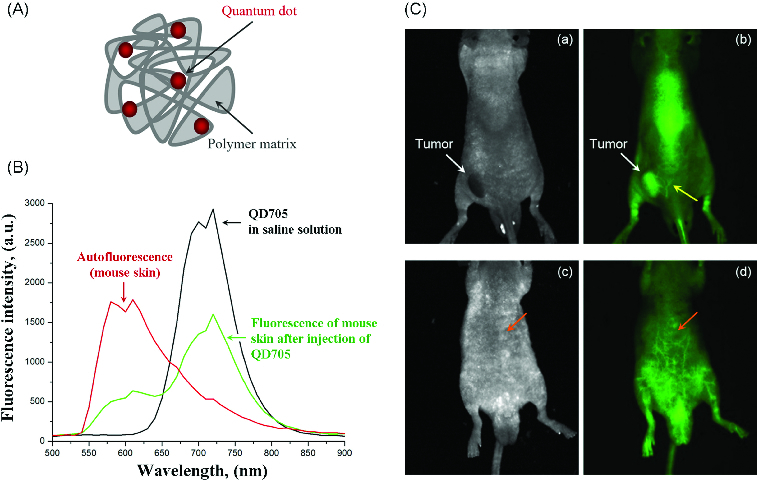
(A) Scheme of quantum dot-labelled polymersomes. (B) Fluorescence spectra of QD^705^ in saline solution (on phantom), fluorescence spectra (autofluorescence) of mouse body detected before injection of QD^705^ and fluorescence spectra of mouse body detected after i.v. injection of QD^705^. (C) Images of colon cancer-grafted mouse, obtained 2 min after i.v. injection of QD^705^-lablelled polymersomes: (a and c) – transmission; (b and d) – fluorescence. Note: Yellow arrow indicates the angiogenesis around the tumour. Orange arrow indicates a deep-tissue imaging of blood vessels.

QD^705^ (Qdot®705 ITK^TM^ carboxyl quantum dots) were purchased from *Invitrogen*. Water-soluble polymersomes were prepared from chemically modified chitosan as it was described by Lee et al. [18]. A schematic structure is shown in Figure 1(A). Labelling of polymersomes with QDs was carried out via carbodiimide chemistry, using N-(3-dimethylaminopropyl)-N′-ethylcarbodiimide hydrochloride (EDC) as a zero-length cross-linker.[19] The nanoparticles were characterized by transmission electron microscopy, dynamic light scattering and fluorescent spectroscopy. QD concentration in polymersomes was calculated by the method of Yu et al. [20].

Isoflurane was purchased from *Abbott* (Japan).

All chemicals used in this study were analytical or HLPC grade.

### Experimental cancer model

*Balb/c nude* mice (21 ± 2 g) were used. *Colon26* cells (1 × 10^5^ in 10 μL PBS, pH 7.4) were inoculated subdermally in the left/right hindpaw. All measurements were performed ∼9–10 days after inoculation, when the tumour size was ∼100 mm^3^.

All experiments were conducted in accordance with the guidelines of the Physiological Society of Japan and were approved by the Animal Care and Use Committee of the National Institute of Radiological Sciences, Chiba, Japan.

### Optical imaging

All experiments *in vivo* were conducted under anaesthesia.

Briefly, the mouse was anaesthetized with 1.5% isoflurane using mask. The tail veil was catheterized for the administration of nanoparticles and the mouse was fixed in the camera of the *Maestro EX Imaging System*, connected to anaesthesia device. The body autofluorescence was registered at excitation filter 435–480 nm and emission filter 700 nm (longpass). Nanoparticles (QD^705^ or QD^705^-labelled polymersomes) were injected intravenously (i.v.) via the tail vain (single dose – 80 nmol; 100 μL volume) and the whole body fluorescence was registered on the back and stomach side at different time-intervals.

The data were analysed by *Living Image In Vivo Imaging* software (*Maestro* version 2.10.0).

## Results and discussion

The first step of the study was to select appropriate concentration of QD^705^ for *in vivo* application, which gives a high signal-to-background ration without saturation of the fluorescent signal and existence of the artefacts. Thus, before fluorescent imaging on cancer-grafted mice, all imaging parameters were selected on the basis of preliminary experiments using QD^705^ solutions in different concentrations, applied on phantoms and healthy mice (subcutaneous [s.c.] and i.v. injections in different volumes).

Figure 1(B) shows the fluorescent spectra of QD^705^ in saline solution (black line), autofluorescence spectra of mouse body (red line) and their overlap after injection of QD^705^ in mouse via the tail vain (green line). The data indicate that QD^705^, emitting in the NIR region of spectra (700–900 nm), allow to overcome the autofluorescence of mouse body and to obtain a strong fluorescent signal coming from the nanoparticles.

The second step of the study was to investigate the possibility to deliver QDs in tumour using polymersomes as nanocarriers.

The images in Figure 1(C) were obtained on colon cancer-grafted mouse, injected with QD^705^-labelled polymersomes in concentration, selected in step one (80 nmol, single i.v. injection). The images were obtained 2 min after the injection of the nanoparticles. In this short period after injection, the tumour was visualized on the basis of angiogenesis. The quantum yield of QD^705^ was high enough to allow a deep-tissue imaging of blood vessels. The yellow arrow in Figure 1(C) (b) indicates the angiogenesis around the cancer. The orange arrow in Figure 1(C) (d) indicates a deep-tissue imaging of blood vessels.

**Figure 2.**
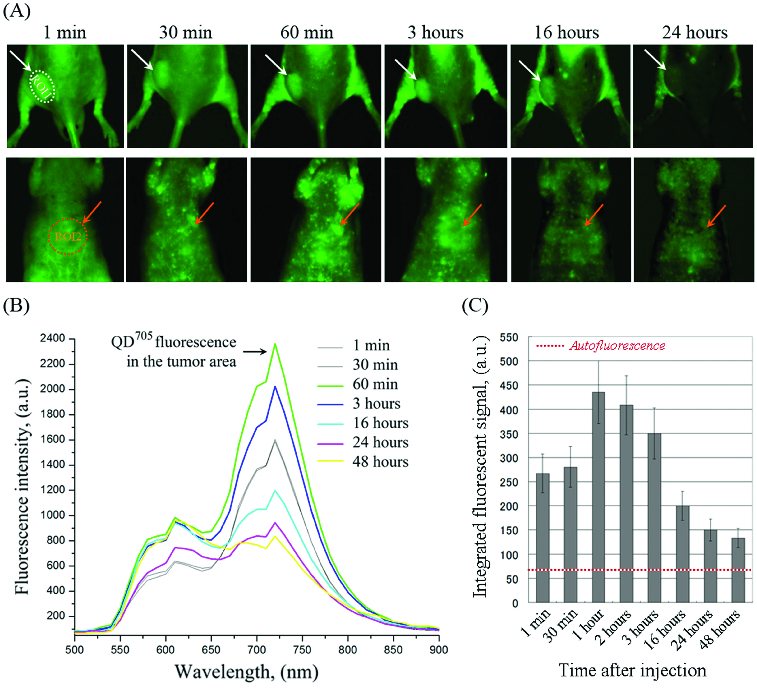
(A) Representative fluorescent images of colon cancer-grafted mouse, obtained at different time-intervals (within 24 hours) after i.v. injection of QD^705^-labelled polymersomes. Regions-of-interest (ROI): ROI-1 – tumour area; ROI-2 – liver area. (B) Dynamics of fluorescent spectra in tumour area (ROI-1), overlapped with autofluorescence spectra of mouse body, obtained within 1–24 hours after i.v. injection of QD^705^-labelled polymersomes. The fluorescent spectra were extracted from the images in (A). (C) Kinetics of QD^705^ fluorescence decay in ROI-1 within 48 hours after injection, calculated at λ_em_ = 705 nm. The data are means ± SD from four animals.

Figure 2 shows the dynamics of QD fluorescence intensity in the tumour (ROI-1) and liver area (ROI-2) of colon cancer-grafted mice. The images were obtained at different time-intervals within 24–48 hours and fluorescent spectra were divided from the regions of interest. In ROI-1, the fluorescence intensity increased within 1–3 hours after injection – a maximum fluorescent intensity was detected on the 1st hour. After that, the fluorescence intensity in ROI-1 decreased, but did not reach the baseline within 48 hours (Figure 2(C)). A very weak QD fluorescence was detected in ROI-2 (liver area). QD fluorescence intensity was detected in the whole body during 48 hours. The data suggest that QD^705^-labelled polymersomes had a long life-time in the bloodstream. They were accumulated into the tumour as a result of enhanced permeability and retention effect. A negligible amount was accumulated into the liver. The data indicate that described polymersomes are appropriate carrier for passive drug delivery in tumours. Small QD fluorescent spots were visualized on the stomach side of the mice, which are probably lymph nodes (Figure 2(A)). It seems that the used polymersomes could also be accumulated into the lymph nodes, which makes them appropriate drug-carrier for treatment of metastases.

**Figure 3.**
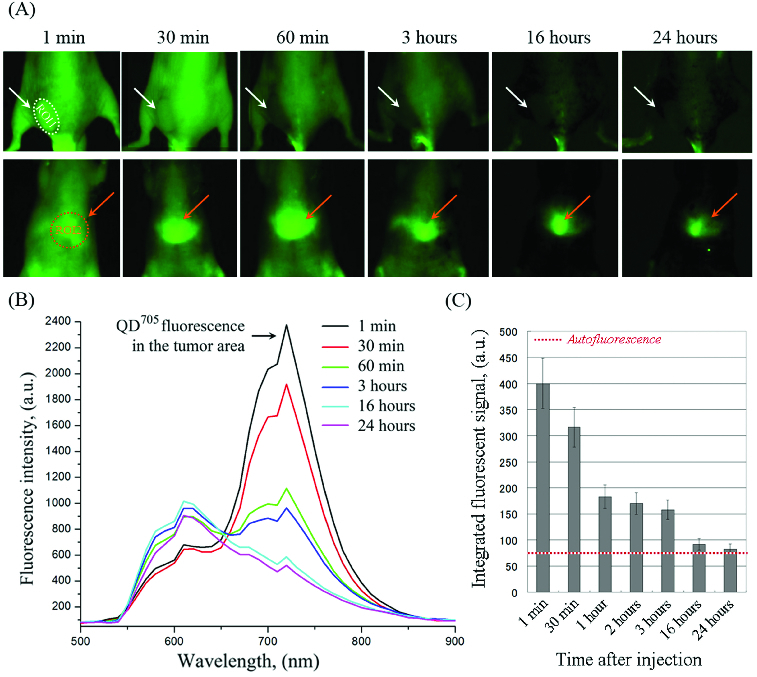
(A) Representative fluorescent images of colon cancer-grafted mouse, obtained at different time-intervals (within 24 hours) after i.v. injection of QD^705^. Regions-of-interest (ROI): ROI-1 – tumour area; ROI-2 – liver area. (B) Dynamics of fluorescent spectra in tumour area (ROI-1), overlapped with autofluorescence spectra of mouse body, obtained within 1–24 hours after i.v. injection of QD^705^. The fluorescent spectra were extracted from the images in (A). (C) Kinetics of QD^705^ fluorescence decay in ROI-1 within 24 hours after injection, calculated at λ_em_ = 705 nm. The data are means ± SD from four animals.

Native QD^705^ nanoparticles were used for comparison (Figure 3). They were accumulated in the liver (Figure 3(A) – ROI-2) and were not accumulated in the tumour (Figure 3(A) – ROI-1). The fluorescence intensity in ROI-1 increased immediately after injection, followed by a rapid decrease to the baseline within ∼16 hours (Figure 3(B) and 3(C)). The tumour was visualized only within 30 min after injection due to angiogenesis.

Recently, several teams have reported *in vivo* application of size-controlled polymersomes, composed of different block copolymers, for image-guided drug delivery in solid tumours on experimental animals.[21–23] Levine et al. [21] have described NIR-emitting polymersomes, composed of poly(ethylene-oxide)-block-poly(caprolactone) and including doxorubicin and their application for optical image-guided therapy of cancer. Muthiah et al. [22] have described polymersomes, composed of polysuccinimide and including magnetic nanoparticles and paclitaxel for MRI-guided drug delivery in tumours. Pourtau et al. [23] have reported antibody-functionalized magnetic polymersomes for *in vivo* targeting and imaging of bone metastasis using high-resolution MRI.

The growing number of studies on polymersomes demonstrates that these nanoparticles are new and valuable tools for theranostic applications. The enhanced stability and tunability of polymersomes will ultimately lead to the development of effective carriers for *in vivo* drug delivery, molecular imaging and cellular mimicry. In drug delivery, the potential to co-encapsulate two drug molecules in the same polymersome enables combination therapies and eliminates the need to individually administer two separate drug formulations.[21]

## Conclusions

The present study suggests that size-controlled long-circulating polymersomes are very promising carrier for drug delivery in cancer and probably in lymph nodes, including delivery of small nanoparticles as quantum dots. The delivery of non-functionalized polymersomes into the cancer is based on the enhanced permeability and retention effect. These nanoparticles have a great theranostic potential.
